# Tyrosine Kinase Inhibitors and Interferon

**DOI:** 10.4084/MJHID.2014.006

**Published:** 2014-01-02

**Authors:** Maria Dimou, Panagiotis Panayiotidis

**Affiliations:** 1^st^ Department of Propaedeutic Medicine, Division of Hematology, University of Athens, Greece.

## Abstract

The use of interferon-a (INF) in chronic myeloid leukemia, when it started in the 80s, was considered as a breakthrough in the therapy of this disease; INF administered alone or in combination with aracytin was the standard choice for treatment for Chronic Myeloid Leukemia (CML) patients unfit for bone marrow transplantation. With the appearance of the first Tyrosine Kinase Inhibitor (TKI) (imatinib) and based on the results of the pivotal IRIS trial, imatinib monotherapy was the new treatment of choice for CML, according to the ELN recommendations. The possibility of combining INF with imatinib, for obtaining better therapeutic responses in CML patients has been already tested and reported. The current challenge is the combined use of second generation TKIs with pegylated –IFN, in order to minimize failures to therapy and increase the number of CML patients with deep molecular responses, who may be able to discontinue lifelong treatment.

## Introduction

The beginning of the use of INF as treatment for CML started at 1983 after a report by Talpaz et al that leukocyte IFN induced cytoreduction in CML.^1^ Other studies confirmed the effect of IFN in CML.^2^,^3^,^5^ Complete hematologic responses were observed in 80% of CML patients treated with IFN and in 7–10% of them complete cytogenetic responses (CCyR) were obtained. CML patients in CCyR under IFN-a treatment have an 80% chance of 10 years survival.^6^,^7^ Since there was a clear benefit in the survival of CML patients treated with IFN compared to bulsufan or hydroxyurea, IFN became the standard of CML therapy in the 90s and early response after 3 months of therapy was associated with good outcome.^8^ The major issues raised during that time were the right dose of IFN-α used and the issue of combining IFN with other drugs, e.g. busulfan, hydroxyurea, aracytin.^2^–^5^ Most studies were performed with high dose of IFN-α, at 5MU/m^2^/day and side effects, mainly flu-like symptoms, fatigue, hematologic toxicity, weight loss, neurotoxicity and depression and cardiotoxicity. Dose reductions in IFN-α were frequent both in clinical trials and the everyday clinical practice. In a study comparing high dose IFN versus low dose (3MU/day/5 das/week) no difference is responses was observed. Overall survival after 5 years was 50% in the low dose arm versus 49% in the high dose IFN arm. Complete cytogenetic response was 9% in the the low dose arm and 7% in the high dose IFN arm.^9^

## Mechanism of Action of IFN–α in CML

CML progenitor stem cells are deficient in adhering to bone marrow stromal cells, when compared to normal hematopoietic stem cells.^10^ This was considered to contribute to the pathogenesis of CML, since it was suggested that circulating CML stem cells, due to their failure to adhere to bone marrow, could contribute to leukocytosis and extra-medullary hemopoiesis e.g. splenomegaly.

A minority of CML patients treated with a-IFN obtained CCyR, and some have discontinued treatment. In a study by Mahon et al. IFNα treated CML patients who were in CCyR or bcr-abl negativity, discontinued IFN and 8 patients lost CCyR after 3–33 months.^11^ Seven patients that were in CCyR>24 months and were bcr-abl negative before discontinuation did not relapse (median time of observation 36 months).

The results of the pivotal IRIS trial documented the superiority of imatinib compared to the combination of IFN +Aracytine in the treatment of newly diagnosed patients with CML in chronic phase.^12^ In 2013 the TKI inhibitors imatinib, nilotinib and dasatinib are approved as first line therapy in CML,^13^,^14^ while bosutinib and ponatinib are approved for use as second/third line therapy in CML.^15^,^16^

The mode of action of TKIs is obviously, totally different from that of IFN and the issue of combining these drugs for the treatment of CML was soon raised by different groups. The basis for these trials was to have better response rates and to obtain deeper, sustained molecular responses. Although deep molecular responses (MR^4^, MR^4.5^) may not have an impact on patient survival, they may offer a higher probability of successful treatment discontinuation of TKIs.

However, all TKIs examined so far, have failed to eliminate the CD34+ CD38− CML stem cells.^17^,^18^ In contrast to mature CML sells, survival of CML stem cells is not dependent on bcr-abl activity and various other pathways participate in CML cells survival; cellular interactions between CML stem cells and bone marrow stroma, activation of several pathways (Wnt, Hedgehog signaling, autophagy, etc).^19^–^22^

## Published Trials of TKIs plus IFN in CML

In a study by the CML Italian group, 76 early phase CML patients were treated with 400 mg imatinib in combination with 50 or 100 or 150 μg/week of pegylated–IFN. The median administered dose of peg-IFN was 32–36 μg/week and 45 of 76 patients (59%) discontinued peg-IFN during the first year of treatment.^23^

In the French SPIRIT trial,^24^ 636 patients with untreated chronic phase CML were randomized to imatinib 400mg, imatinib 600mg, imatinib 400mg plus peg IFN 90μg/week, and imatinib 400mg plus cytarabine. The dose of cytarabine was 20mg/m^2^ on days 15–28 of a 28 days cycle. During the trial, the arm of imatinib 600 mg/day and the imatinib plus aracytin arm was omitted due to toxicity and the superior results of imatinib plus peg-IFN. The dose of peg-IFN after the first year of the study was reduced to 45μg/week which was better tolerated by the patients. In the first year of the trial, 45% of patients randomized to the peg-IFN arm discontinue treatment. The major side effects in this group of patients was an increase in hematological toxicity, skin rash and asthenia, compared to the group that received imatinib alone. The median dose of peg-IFN delivered in the first year of the study was 54μg/week. No difference in the rate of cytogenetic response after 12 months of therapy was noted between the four arms of the study. In patients who managed to receive peg-IFN more than 12 months, 82% had MMR and 49% had MR4 after two years compared to 43% and 21% respectively in the imatinib 400 arm. Patients who manage to receive IFN for less than 4 months had significantly inferior results. The results of this study demonstrated that the most tolerated dose of pegylated IFN is around 45μg/m^2^ and the combination with imatinib results in faster and deeper molecular responses.

The rate of responses in the Imatinib-peg-IFN arm was comparable to the responses obtained with the second generation inhibitors nilotinib and dasatinib.^15^,^16^ In the German CML IV randomized study,^25^ 1104 newly diagnosed CML patients in chronic phase were randomized to receive imatinib 800mg/d (n=338), imatinib 400mg/d (n=325), or imatinib 400mg/d plus interferon alfa (IFN- α; n=351). Primary endpoint of the study was MMR at 12 months.

MMR rates were similar between the imatinib 400 mg and the arm of imatinib 400+IFN-α (44% vs 46%). The IFN-α used in the study was the classical recombinant IFN –a at a schedule of 3 million units × 3 times weekly s.c. It is possible that the IFN-α formulation and e.g. pegylated versus classical recombinant may and dosage of IFN used may be responsible for the different therapeutic results between the CML IV and the SPIRIT trial.

In another phase II study,^26^ the Nordic group randomized 112 chronic phase CML patients with low or intermediate risk who were in complete hematologic remission after therapy with 400mg imatinib to a) continuation with imatinib alone b) receive imatinib plus 50μg pegylated IFNa/week; 34 of the 56 patients in the combination arm discontinued peg-IFNα and the dose of peg-IFN was reduced to 30μg/week. In accordance with the results obtained in the SPIRIT trial, the MMR rate at 12 months was significantly higher in the imatinib plus Peg–IFN-α arm (82%) compared with the imatinib monotherapy arm (54%).

In a trial by the MD Anderson group, 94 early phase CML patients were randomized in two arms a) 800mg of imatinib and b) imatinib 800mg plus 0,5μg/kg peg-IFN/week and GM-CSF 125mcg/m^2^ three times per week subcutaneously. No difference in MMR at 12 months or at any other time point during the study was observed. It should be noted, however, that peg-IFN-α was discontinued in all patients due to side effects or other reasons.^27^

IFN-α has been recently reported to induce proliferation of hematopoietic stem cells in mice.^28^ Induction of CML stem cells to exit G0 and entry into cell cycle may render them susceptible to TKIs and produce the superior results when the two agents are combined for the treatment of CML patients. CML stem cells have been shown to be resistant to the administration of the second generation inhibitors dasatinib and nilotinib.^18^,^19^ Concomitant administration of IFN-α may increase the proportion of CML patients achieving deep molecular responses that may lead to treatment discontinuation.

A number of clinical trials are currently exploring the combination of IFN-α with TKIS in the treatment of CML (Table 1).

In trial No 1 (NICOLI), the maximum tolerated dose of peg-IFN-α in CML patients receiving nilotinib will be determined.

In trial No 2, the aim is to investigate whether patients with chronic-phase chronic myeloid leukemia who have achieved a CCyR) on imatinib or nilotinib can then be treated with a combination of the tyrosine kinase inhibitor and peg-IFN-α for 2 years. Subsequently these patients will have their therapy discontinued. Relapse-free survival (RFS) rate 1 year after discontinuation of the TKI and IFN will be the main objective of the study.

Forty CML patients will participate in this study. The planned dose for peg-IFN-α, is 150μg s.c./week, and many side effects and drug discontinuations are expected.

In the trial No 3, (NILOPEG), 60 chronic phase CML patients will receive nilotinib 300mg twice a day +Pegylated interferon 2a, 45μg weekly. Peg-IFN will start after 2–12 months according to investigator choice. Primary end point is the “CMR” rate at 12 months.

In the the trial No 4, by the Nordic group, the purpose will be to assess the effect of switching CML patients, who have been treated with imatinib ≥ 2 years and who have stable detectable molecular residual disease between 0.01–1.0 percent (IS), to a combination of Nilotinib and PegIFN-α, in terms of the proportion of patients who achieve confirmed MR4.0. Patients will start therapy with 300 mg nilotinib BID for 3 months, and then Peg IFN will be added at 25μg/week s.c. In the absence of toxicities, the dose will increase to 40 μg/week s.c. The estimated enrollment is 60 patients.

In the study No 5, by the Nordic group, 35 newly diagnosed CML patients will receive dasatinib 100 mg/day for 3 months, and then 15 μg/week Peg-IFN. If no significant toxicities emerge, peg-IFN-α dose will escalate up to 25μg/week for the next 9 months. Primary end point is the rate of MMR after 1 year.

The study No 6, (TIGER) by the German group, 652 chronic phase CML patients will be randomized to:

- nilotinib 300mg bid plus Peg-IFN- α 30μg/week s.c. After confirmed MMR and at least 24 months therapy, nilotinib will be discontinued. When MR4 for more than a year, discontinuation of peg-IFN-α.
- nilotinib 300mg bid for more than 3 years. If MR^4^ more than a year, discontinuation

The aim of the trial is to improve treatment strategies in CML by improving induction therapy and deescalating maintenance therapy, using low-dose IFN as inducer of immune surveillance.

Primary end point will be the rate of MMR after 18 months and feasibility to discontinue therapy when patients have in stable MR^4^ response for more than a year.

From the current trials incorporating IFN in the therapy of CML, it is obvious that the pegylated form of IFN is universally used, at doses much lower than the ones used in previous studies. The question is if these lower peg-IFN doses with acceptable toxicities will retain their activity against CML. The possibility of effective targeting the CML stem cells via the combination of TKIs and peg-IFN remains an open question. If this combination will succeed in inducing higher rates of treatment discontinuations then IFN-a (the pegylated form) will reappear officially in the treatment of CML patients.

## Figures and Tables

**Table 1:** 

CLINICAL TRIALS OF IFN +TKIs				
	ClinicalTrials.gov Identifier:	Phase of study	No of Patients	Official title
1	NCT01220648 Active,	IV	18 Years and older	An Open Label, Nonrandomized, Single-center, Phase I Trial of Pretreated Philadelphia Chromosome Positive (Ph+) Chronic Myeloid Leukemia Patients in Chronic Phase (CML-CP) With Nilotinib in Combination With Low Dose Interferon-alpha (IFN) - NICOLI Study–
2	NCT00573378 Recruiting	II	40 pts 18 Years and older	A Combination of Imatinib or Nilotinib Together With Pegylated Interferon-α2b in Chronic-Phase Chronic Myeloid Leukemia: A Phase II Pilot Study Targeting Both the Primitive and Differentiated CML Progenitor Populations
3	NCT01294618 This study is ongoing.	II	60 18 Years and older	“Phase II Multicenter Study Evaluating the Efficacy and the Safety of a Combination of Nilotinib Plus Pegylated Interferon Alpha 2a for de Novo Chronic Phase Chronic Myelogenous Leukemia Patients” (NILOPEG)
4	NCT01866553 Active	II	60 pts 18 Years and older	A Phase II, Single Arm, Multicenter Study of Nilotinib in Combination With Pegylated Interferon-α2b in Patients With Suboptimal Molecular Response or Stable Detectable Molecular Residual Disease After at Least Two Years of Imatinib Treatment (NordDutchCML009)
5	NCT01725204 Recruiting	II	35 pts 18 Years to 70 Years	A Safety and Efficacy Study of Adding Low Dose Pegylated IFN- alpha 2B to Standard Dose Dasatinib in Patients With Newly Diagnosed Chronic Phase Myeloid Leukemia
6	NCT01657604 Recruiting	III	652 pts 18 Years and older	Treatment Optimization of Newly Diagnosed Ph/BCR-ABL Positive Patients With Chronic Myeloid Leukemia (CML) in Chronic Phase With Nilotinib vs. Nilotinib Plus Interferon Alpha Induction and Nilotinib or Interferon Alpha Maintenance Therapy (Tiger study)
